# Adapting Child Development Assessment Tools to the Rural Indian Context

**DOI:** 10.3390/children11091115

**Published:** 2024-09-12

**Authors:** Riti Chandrashekhar, Baldeep K. Dhaliwal, Ananya Rattani, Rajeev Seth, Suba Guruprasad, Himani Khanna, Anita Shet

**Affiliations:** 1 Department of International Health, Johns Hopkins Bloomberg School of Public Health, Baltimore, MD 21205, USA; bdhaliw1@jhu.edu (B.K.D.); ashet1@jhu.edu (A.S.); 2 Bal Umang Drishya Sanstha, New Delhi 110016, India; 3 International Vaccine Access Center, Johns Hopkins Bloomberg School of Public Health, Baltimore, MD 21205, USA; 4 St. George’s University Hospital NHS Foundation Trust, London SW17 0QT, UK; 5 Continua Kids, New Delhi 110034, India; himani@continuakids.com

**Keywords:** adaptation, developmental tests, cognitive tests, rural research

## Abstract

Background: Child development assessment tests serve many purposes, including educational placement, identifying cognitive weaknesses, and initiating early interventions. Much of the research associated with developmental testing has been conducted in high-income countries, offering limited guidance on adapting these tests to other settings. Objectives: As part of the first phase of a study exploring the impact of childhood vaccines on cognition and school attainment, we aimed to assess the feasibility of enrolling children from the community, documenting immunization, and conducting child development assessments for children between 18 months and 8 years of age in a rural setting in Haryana, India. Methods: To ensure assessments are optimally tailored to the context, child development assessment tests require valid translation and cultural adaptation. This report describes the rigorous seven-step adaptation process we designed for the contextually appropriate adaptation of the following three child development assessment tests: the Bayley Scales of Infant Development-IV, the Stanford Binet Intelligence Scale Fifth Ed. for Early Childhood, and the Wechsler Intelligence Scale for Children IV. Results: This process involved translating tests into the local language, back-translating them for accuracy, adapting them to the rural context via several iterations, and field-testing to refine and validate adaptation quality. Conclusions: This adaptation process may be beneficial for other researchers involved in adapting child development assessment tests to other settings. Further, this adaptation process may inform other researchers involved in adapting tests for diverse settings.

## 1. Introduction

Cognitive function refers to a range of abilities, including abstract reasoning, processing information, manipulating objects, encoding data, and retrieving information [1]. Childhood development, particularly cognitive development, has significant impacts across an individuals’ lifespan [2]. In recent years, there has been an increased focus on reviewing developmental trajectories across childhood years, as skills and trajectories acquired in childhood can have long-term impacts on an individual [3]. Delays in developmental milestones during childhood can have economic repercussions, including the potential to contribute to the intergenerational perpetuation of poverty [4].

There is compelling evidence that the early detection of developmental delays, followed by early intervention and appropriately tracked long-term management, is critical for a child’s developmental health [5]. For instance, a study by Walker et al. (2011) [5] found that inadequate cognitive stimulation is a risk factor during childhood as it interferes with brain development. Child development assessment tests include a series of tasks that gradually increase in complexity to assess if a child meets age-appropriate developmental milestones. However, these tests have traditionally been developed within narrow geographic high-income settings, primarily scaled to North American and European children [6], revealing a substantial gap with tests specifically designed to assess childhood milestones in low- and middle-income countries (LMICs) [7]. While studies on developmental trajectories in LMICs have estimated that 200 million children fail to reach their developmental potential [4], many of these countries use child development assessment tests that have been standardized in Western or high-income settings. Although these tests have had several iterations over decades of research, these iterations have consistently been made for children in high-income countries, such as the United States. This could lead to results that may not reflect the true cognitive abilities of children living in LMICs [3].

Current ‘standardized’ tests have typically been developed on a sample which may be diverse within a country, but not necessarily representative across populations in other countries, thus limiting their ability to detect milestones appropriately in a global setting [3]. It has been previously established that cognitive tests tend to be bound by cultural contexts and social norms [8,9]. The Stanford Binet Intelligence Scale, for example, has been tested for validity based on cultural references relevant to European and North American cultures (e.g., drinking tea out of a teacup with a saucer, or bathing in a bathtub). Although these practices are common in Europe and North America, they are not regularly seen in a South Asian context. While languages and dialects have nuances that need to be considered while adapting cognitive tests, cultural practices in child development assessment tests also need consideration for validation purposes [10].

To overcome the limitations of standardized child development assessment tests within LMIC settings, the following two solutions have been suggested: culturally and literally adapting existing validated developmental tests, or assembling new locally designed contextually relevant developmental tests [11,12]. While adapting an existing test is a common practice [12,13], many assessments require significant iterative adaptive steps for use in LMIC settings [12]. Child development assessment tests tend to be bound by cultural contexts, social norms, and the nuances of language and dialects, requiring careful adaptation to the local context [9]. Assembling new child development assessment tests is a laborious and expensive prospect, hindered by limited access to quality education and healthcare resources in LMICs. These limitations can potentially lead to disparities in test performance and reliability. To avoid these glaring limitations, we chose to adapt existing child development assessment tests for young children. 

Our adaptation process aims to fill a gap in the literature by demonstrating how this approach can benefit other researchers conducting child development assessments in LMICs and serve as a guideline for adapting broader tests in diverse settings.

## 2. Materials and Methods

### 2.1. Setting

We completed the child development assessment tool adaptation process in the Nuh district in the North Indian state of Haryana. This region has poor educational indicators, with only 44% of women and 60% of men having ten or more years of schooling [14]. We led these efforts in Ghasera, the largest village, as well as other neighboring villages in Nuh district. Each of these villages has multiple centrally located Anganwadi (basic rural preschool educational) centers. These villages were selected as field staff have strong relationships with members of these communities. Further, the village Anganwadi centers, where research assistants planned to pilot and implement these tests, were accessible to participants. All testing was conducted in Anganwadi centers in the presence of well-known field workers to facilitate a comfortable environment for participating children.

### 2.2. Child Development Assessment Tests

Our team initiated the adaptation process by following the method used by researchers who modified the Wechsler Intelligence Scale for Children IV (WISC-IV) for a population in Vietnam [15]. The child development assessment tests that we adapted were the (a) Bayley Scales of Infant Development-IV (BSID-IV), (b) Stanford Binet Intelligence Scale Fifth Ed. for Early Childhood (SB5 Early), and (c) the India adaptation version of the Wechsler Intelligence Scale for Children IV (WISC IV). These tests were selected as they are conceptually broad, covering a wide intellectual area; have been used globally to facilitate cross-cultural research; and, collectively, these tests span the wide age range, from 18 months to 8 years, relevant to our parent study.

#### 2.2.1. Bayley Scales of Infant Development-IV

The first version of the Bayley Scales of Infant and Toddler Development (BSID) was developed and published in the 1960s [16], and it has been revised multiple times [17]. The BSID-IV, published in 2019, focuses on early childhood assessment, assessing physical, cognitive, social-emotional, linguistic, and behavioral milestones. We selected this test as it allowed us to test 18–35-month-olds, and the time commitment required for this test was lower than older versions of the BSID [17].

#### 2.2.2. Stanford Binet Intelligence Scale Fifth Ed. for Early Childhood

The Stanford Binet Intelligence Scale Fifth Ed. (SB5) measures the following five cognitive factors across verbal and non-verbal domains: quantitative reasoning, visual–spatial processing, working memory, and fluid reasoning [18]. We chose SB5 Early, the version best suited for children between 24 months and 5 years and 11 months.

#### 2.2.3. Wechsler Intelligence Scale for Children IV

The Wechsler Intelligence Scale for Children IV Indian adaptation was used [19] after it was adapted from English to Hindi. The WISC-IV Indian adaptation was chosen for this study as it had the most relevant examples for the participants, and it would require the fewest adaptations to be tailored to the setting (i.e., the use of the metric system).

### 2.3. Adaptation Process

**Figure 1:**
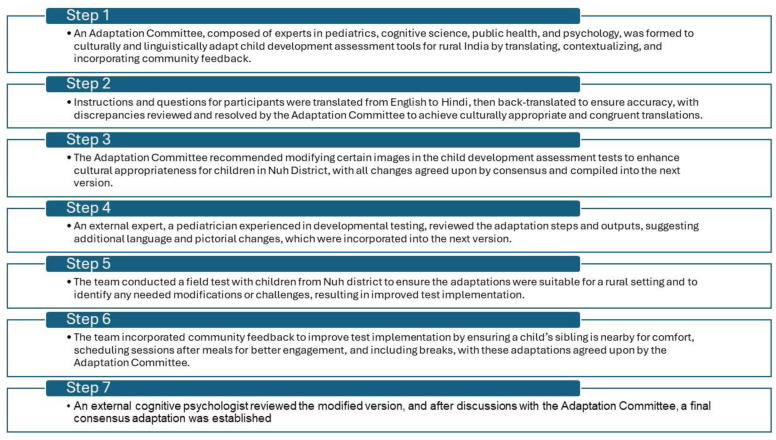
Adaptation process of child development assessment tests.

To adapt these tests, we refined the approach of Dang et al. [15] to a seven-step process, best tailored to our context (Figure 1). This process began by forming an adaptation committee, translating tests into Hindi while accounting for local language and dialects, back-translating tests to English for accuracy, adapting pictures, completing an external expert review, leading field tests, soliciting community input, and conducting a second external review to reach a final consensus and perform implementation (Figure 1).

#### 2.3.1. Step 1: Adaptation Committee Formation

Before initiating the adaptations of the child development assessment tools, an Adaptation Committee was formed. The members of the committee comprised two general pediatricians, a developmental pediatric specialist, a cognitive science researcher, a public health researcher, and two research assistants trained in applied psychology. Committee members were selected based on their training and experience in rural and urban Indian settings, their familiarity with psychometric testing, and their cultural and linguistic abilities in rural India. The committee members worked in tandem with the Nuh field staff during the adaptation process to rapidly incorporate feedback from local community members. The Adaptation Committee independently documented the items in the developmental tests that needed to be translated from English into Hindi, and they noted aspects of the tests that needed contextualization. The meetings of the Adaptation Committee focused on reaching a consensus among members on the areas to be considered for adaptation and translation. Attendees at these meetings also discussed the need for changes in the following aspects: toys associated with tasks, activities, pictorial representations for relevancy, and cultural adaptations. The final adaptations are detailed in the Supplemental Material File.

#### 2.3.2. Step 2: Child Development Tests Translation

All instructions and questions directed at participants were translated from English into Hindi by a research assistant. Once translations were compiled, the Hindi versions of the instructions and questions were shared with a different research assistant who back-translated the Hindi versions into English. Both research assistants were fluent in English and Hindi, and they had prior experience with the translation and back translation of psychometric testing. Based on discrepancies between Hindi translations and back translations, the research assistants made additional edits. Once the first phase was completed, the first versions of the translations were circulated to the full Adaptation Committee for a review of their cultural appropriateness (v 1.0). The pediatricians and cognitive scientists of the Adaptation Committee compared the back-translated version with the original translation to ensure translation congruity. Identified discrepancies were resolved through a consensus meeting with all members of the Adaptation Committee. Subsequent Adaptation Committee meetings were held after the translation and adaptation of tests to review and assess the modifications. Discrepancies in the back-translated versions were flagged during the pilot testing and changes were made to produce a consolidated version for a full Adaptation Committee review (v 2.0). Final adaptations are detailed in the Supplemental Material File.

#### 2.3.3. Step 3: Child Development Tests Translation

**Figure 2:**
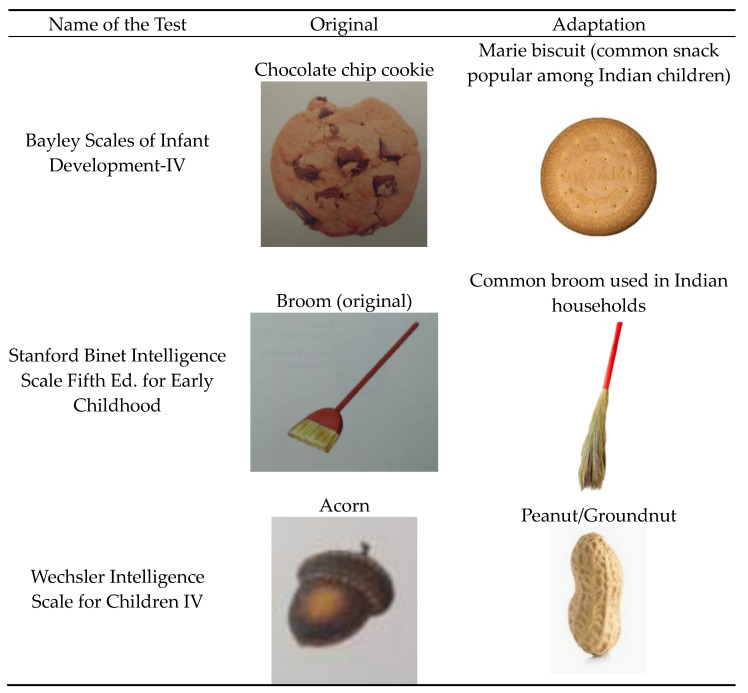
Examples of cultural adaptations made in child development assessment tests.

The adaptation committee recommended that certain images that appeared in subtests of the child development assessment tests should be changed to improve cultural appropriateness and facilitate understanding by children in Nuh district (Figure 2). Each suggested adapted picture was similar to the original, and these pictures were selected based on Adaptation Committee consensus. The translated and adaptive changes were compiled to produce the next version (v 3.0).

#### 2.3.4. Step 4: External Expert Review

Our team consulted with an external independent expert to review the adaptation steps and outputs. This expert was a practicing pediatrician within the community who had prior training in conducting developmental tests in children. The expert reviewed and suggested changes including additional language and pictorial changes, which were incorporated (v 4.0).

#### 2.3.5. Step 5: Field Testing

Our team performed a field test to ensure that adaptations were appropriate for a rural setting, and specifically appropriate for children in Nuh district. Moreover, the field test provided the opportunity to uncover potential challenges faced when testing in low-resource settings. During the field test, the researchers aimed to review (a) any non-contextual references (pictorial or verbal) that needed to be modified, (b) the relevance of pictorial adaptations, and (c) the translations of instructions and questions. Research assistants conducted a field test on three children from the same community in age-appropriate test groups. No personal identifiers were included, and monitoring was conducted through direct observation and using detailed notes taken by the research assistants during and after the field testing sessions. The researchers used these data to evaluate the children’s understanding and engagement with the adapted materials, noting any confusion or lack of comprehension. The team analyzed these notes, focusing on identifying specific patterns and instances where children struggled with the test materials. Researchers categorized these issues into broader categories (i.e., language barriers, cultural mismatches, and practical difficulties). They also assessed the effectiveness of the adapted pictorial elements and the translations of instructions and questions, noting any areas where further modifications may be needed to enhance comprehension and cultural relevance. The field tests provided insights on aspects of the test which needed changes, but also provided insights on more appropriate ways to implement tests. For example, some pictorial references were found to be culturally irrelevant or confusing to the children, leading to adjustments in the images used. In addition, the field test revealed the importance of using simple, clear language and providing examples relevant to the children’s everyday experiences. These findings informed the subsequent rounds of adaptation, ensuring that the tests were both culturally appropriate and practically feasible for use in rural, low-resource settings.

#### 2.3.6. Step 6: Community Input

Our team also sought community input on the implementation processes of the tests. Based on this feedback, our team determined that it was essential to have a child’s sibling nearby for comfort, as opposed to a caregiver, which may be distracting to a child. We also determined that it was important for children to come after a meal for optimal attention and engagement, and that scheduling breaks was similarly essential. The findings of the field test, along with community input and proposed adaptations, were included, and the Adaptation Committee reached a consensus on the translation and picture modifications in the modified version (v 5.0).

#### 2.3.7. Step 7: Second External Review and Final Consensus

This modified version was reviewed by a cognitive psychologist who was external to the Adaptation Committee. After a thorough review and discussion with the Adaptation Committee, a final consensus adaptation was established (v 6.0).

#### 2.3.8. Step 8: Implementation of Adapted Child Development Assessment Tests

Twenty participants were tested using the adapted version of the BSID-IV. Ten participants (aged from 36 months to 5 years) were tested using the adapted SB5 Early, and twenty participants (aged from 6 to 8 years) were tested using the adapted version of the WISC IV.

## 3. Lessons Learned

This report serves as a repository of the process of adaptation of the following three widely used cognitive tools across infant, toddler, and early school-going populations: (a) Bayley Scales of Infant Development-IV, (b) Stanford Binet Intelligence Scale Fifth Ed. for Early Childhood, and (c) Wechsler Intelligence Scale for Children IV. Through this process, we developed an iterative approach which can be replicated and scaled up across global settings.

### 3.1. Iterative Approach to Adaptation

Leveraging an iterative approach to the adaptation process was crucial to ensuring the effectiveness of the selected tests. Through this iterative approach, our team continued to test, refine, and re-test, incorporating feedback and new learnings at each stage of our seven-step process. Our process involved forming a dedicated committee of experts in the Adaptation Committee who meticulously refined the tests multiple times to address shortcomings, two rounds of external review, and community input. This repeated cycle of incorporating feedback and refining the child development assessment tests helped us ensure that the tests were both scientifically sound, as well as practical and relevant to our local context. Using a rigorous iterative approach is critical to continuous improvement and innovation, leading to more robust and appropriate development tests.

### 3.2. Multipronged Approach to Adaptation

Collaboration across a range of individuals, including experts on the Adaptation Committee, external researchers, and local community members, is critical for appropriately adapting child development assessment tests to be best suited to the local context. The experts on the Adaptation Committee brought forth specialized knowledge in developmental psychology and child health, ensuring the assessments were scientifically sound. External researchers were essential, applying rigorous methodologies to assess the validity of the tests. Community members provided practical insights and feedback, ensuring the adapted assessments were relevant and applicable in real-world settings. This collaborative approach ensured that the adapted tests were comprehensive, culturally sensitive, and tailored to meet the diverse needs of children in the particular area. By integrating this wide range of diverse perspectives, adapted assessments can be more effectively tailored to the local context.

### 3.3. Adaptations of Existing Country-Specific Tests

Although there have been Indian adaptations of child developmental assessment tests, many of these may not be applicable to all settings as the selected area may have updated norms and standardizations [20]. Specifically, the Indian adaptations of the tests our team selected were not applicable as they were updated several decades ago, local norms have changed, and they were not designed to account for a rural, low-resource context [20]. As such, it is critical to consider the hyper-local context in which an assessment test will be implemented, even when such a test has been previously adapted to the country. This is essential to ensuring that the assessments encompass language, images, and texts in ways that are best suited to the specific population for which the test is being designed.

### 3.4. Limitations

Our approach was not without limitations. Appropriately translating and assessing the letter sequence in an alphabet proved challenging in Hindi, which is unlike the sequential letter arrangement in the English alphabet. Next, participant responses to cognitive items across tests were diverse based on education levels, particularly among children in lower socioeconomic strata, making it challenging to develop questions that could be applied across the villages. We also observed that children appeared to have ‘survey fatigue’ with repeated piloting, becoming tired or overwhelmed by the length of the developmental tests; hence, the validation of tests had to be performed in stages. Further, as this was a low-resource setting, many children had not previously been exposed to the wide range of objects that are used in the selected tests. As such, children may have been focused on the objects, as opposed to listening to instructions. Ensuring that children are given ample time to play with the objects, prior to initiating piloting, may have helped reduce distractions. Additionally, since this was a pilot study, our aim was to use a method that would function well contextually for the within-population comparison for our study. Hence, we considered several steps—including but not limited to translation, back translation, and letter and picture adaptations—without external validation. Lastly, we recognize that while the current adaptation process is well-suited for this particular low-income setting, there may be additional considerations or steps needed to make it more universally applicable or robust. For future use, validation and standardization would be necessary in order to compare scores with other populations or standards. This approach could help us identify how to further adapt or expand to other contexts to refine our seven-step process further.

## 4. Conclusions

There have been multiple approaches to adapting child development assessment tests to a particular setting. We believe that our seven-step process incorporates the elements needed to facilitate a highly contextualized adaptation process. Our iterative approach takes multiple perspectives into account including perspectives from the research team, from external reviewers, cognitive experts, and, importantly, from the community. As this adaptation process followed a rigorous methodology, we believe that this approach may be beneficial and replicable for other researchers who may be involved in adapting child development assessment tests for other settings.

## Data Availability

No new data were created or analyzed in this study. Data sharing is not applicable to this article.

## Supplementary Materials

The following supporting information can be downloaded at: https://www.mdpi.com/article/10.3390/children11091115/s1, Supplementary Part S1 Bayley Scales of Infant Development-IV (BSID-IV); Supplementary Part S2.

## References

[1] Tucker-Drob E.M. (2019). Cognitive Aging and Dementia: A Life Span Perspective. Annu. Rev. Dev. Psychol..

[2] Tucker-Drob E.M., Briley D.A. (2014). Continuity of genetic and environmental influences on cognition across the life span: A meta-analysis of longitudinal twin and adoption studies. Psychol. Bull..

[3] Sabanathan S., Wills B., Gladstone M. (2015). Child development assessment tools in low-income and middle-income countries: How can we use them more appropriately?. Arch. Dis. Child..

[4] Grantham-McGregor S., Cheung Y.B., Cueto S., Glewwe P., Richter L., Strupp B. (2007). Developmental potential in the first 5 years for children in developing countries. Lancet.

[5] Walker S.P., Wachs T.D., Grantham-McGregor S., Black M.M., Nelson C.A., Huffman S.L., Baker-Henningham H., Chang S.M., Hamadani J.D., Lozoff B. (2011). Inequality in early childhood: Risk and protective factors for early child development. Lancet.

[6] Abubakar A., van de Vijver F.J.R., Abubakar A., van de Vijver F.J.R. (2017). How to Adapt Tests for Sub-Saharan Africa. Handbook of Applied Developmental Science in Sub-Saharan Africa.

[7] Ertem I.O., Dogan D.G., Gok C.G., Kizilates S.U., Caliskan A., Atay G., Vatandas N., Karaaslan T., Baskan S.G., Cicchetti D.V. (2008). A Guide for Monitoring Child Development in Low- and Middle-Income Countries. Pediatrics.

[8] Abessa T.G., Worku B.N., Kibebew M.W., Valy J., Lemmens J., Thijs H., Yimer W.K., Kolsteren P., Granitzer M. (2016). Adaptation and standardization of a Western tool for assessing child development in non-Western low-income context. BMC Public Health.

[9] Tanveer S., Croucher M.J., Porter R. (2022). Cultural modification of neuropsychiatric assessment: Complexities to consider. BJPsych Open.

[10] Waheed W., Mirza N., Waheed M.W., Malik A., Panagioti M. (2020). Developing and implementing guidelines on culturally adapting the Addenbrooke’s cognitive examination version III (ACE-III): A qualitative illustration. BMC Psychiatry.

[11] van de Vijver F., Tanzer N. (1997). Bias and Equivalence in Cross-Cultural Assessment: An Overview. Eur. Rev. Appl. Psychol..

[12] Fernald L.C.H., Kariger P., Engle P., Raikes A. (2009). Examining Early Child Development in Low-Income Countries: A Toolkit for the Assessment of Children in the First Five Years of Life.

[13] van de Vijver F.J.R. (2003). Principles of Adaptation of Intelligence Tests to Other Cultures. Culture and Children’s Intelligence.

[14] Government of India (2021). National Family Health Survey (NFHS-5) 2019-21.

[15] Dang H.-M., Weiss B., Pollack A., Nguyen M.C. (2011). Adaptation of the Wechsler Intelligence Scale for Children-IV (WISC-IV) for Vietnam. Psychol. Stud..

[16] Bayley N. (1965). Comparisons of Mental and Motor Test Scores for Ages 1-15 Months by Sex, Birth Order, Race, Geographical Location, and Education of Parents. Child Dev..

[17] Balasundaram P., Avulakunta I.D. (2022). Bayley Scales Of Infant and Toddler Development. StatPearls.

[18] Horn Newton J., Zeigler-Hill V., Shackelford T.K. (2020). Stanford-Binet Intelligence Scale. Encyclopedia of Personality and Individual Differences.

[19] Wechsler D. (2003). Wechsler Intelligence Scale for Children^®^—Fourth Edition (Wisc-IV) India Complete Kit.

[20] Roopesh B.N. (2020). Review Article: Binet Kamat Test of Intelligence: Administration, Scoring and Interpretation—An In-Depth Appraisal. Indian J. Ment. Health.

