# Supplementary material for: Adapting Child Development Assessment Tools to the Rural Indian Context
Source: Children (Basel). 2024 Sep 12;11(9):1115. doi: 10.3390/children11091115 (PMC11430156; doi:10.3390/children11091115)
Supplement: Supplementary file 1 [file children-11-01115-s001.zip › children-3193300-supplementary.pdf]

Supplementary Part S1

Bayley Scales of Infant Development-IV (BSID-IV)

Section 1: Cognitive

Item	Change required/not required reason
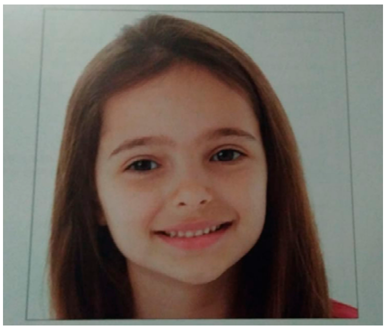	This is Jamie<br><br>Jamie is a two-syllable name. The closest Indian alternatives to the name Jamie which start with the letter J and are two syllables in nature are as follows:<br><br>Jaini<br>Jaanvi<br>Jasvi
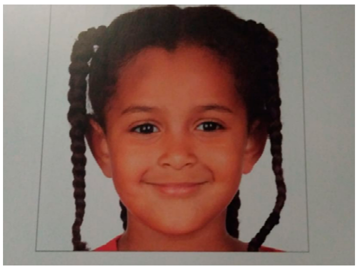	This is Lee<br><br>Lee is a one-syllable name. The closest Indian alternative to the name Lee which start with the letter L and are two syllables in nature are as follows:<br><br>Lia
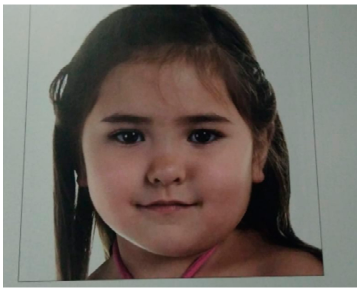	This is Maria<br><br>Changed to this is Mallika

Section 2: Receptive Communication

Item No.	Item	Change required/not required reason
	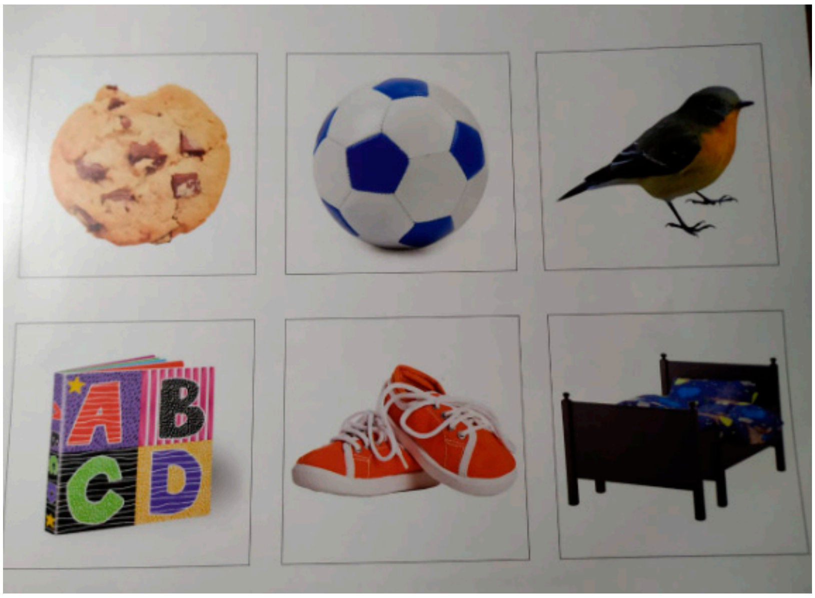	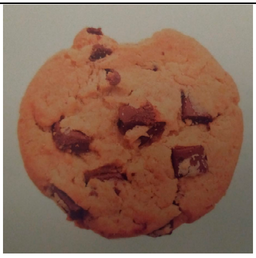<br>Change to<br>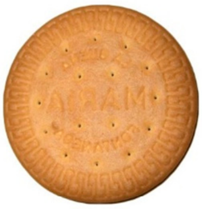

Section 3: Expressive Communication

Item No.	Item	Change required/not required reason
	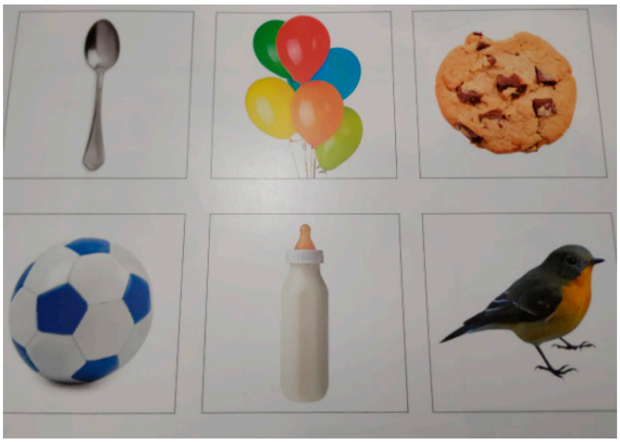	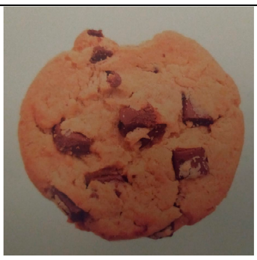<br>Change to<br>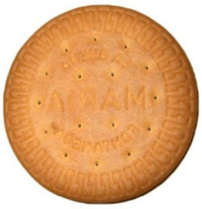

Stanford Binet Intelligence Scale Fifth Ed. for Early Childhood (SB5 Early),

Stanford Binet Item Book 2

Stanford Binet Item Book 2

- **Section: Nonverbal Level 3: Knowledge**

Item No.	Item and Item Description	Change required/not required reason
	The child has to demonstrate how the things work in the images by demonstrating<br>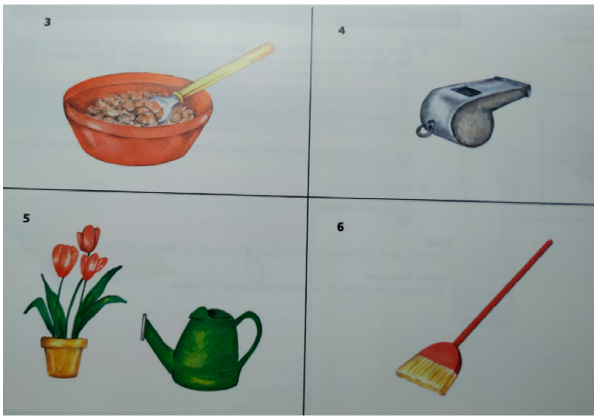<br><br>The child has to demonstrate how the things work in the images by demonstrating	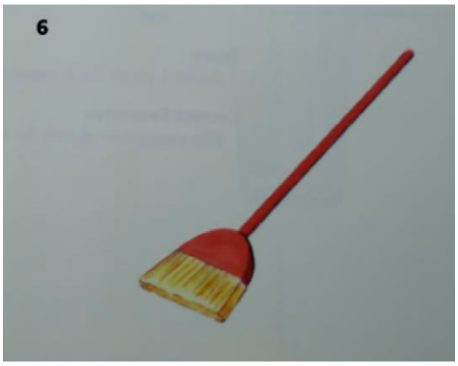<br><br>Change to<br>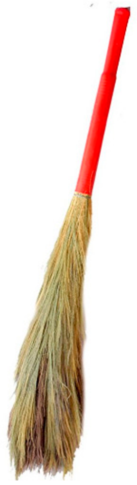

Stanford Binet Item Book 2

- **Section: Nonverbal Level 4: Knowledge**

<table>
<tr><th>Item No.</th><th>Item and Item Description</th><th>Change required/not required reason</th></tr>
<tr><td>Items 1 to 6</td><td>In the following items the child is required to figure and explain the errors in the following pictures:
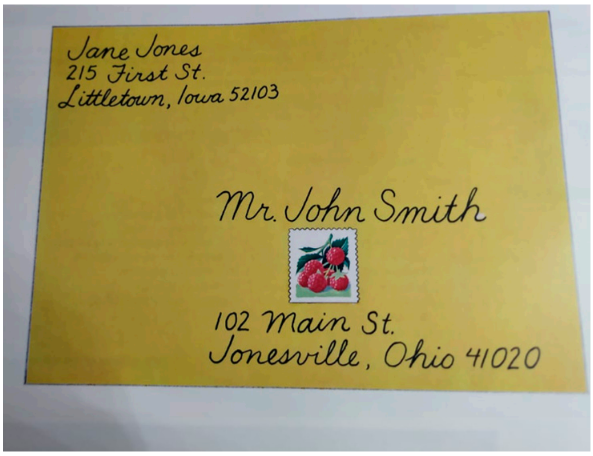
</td><td>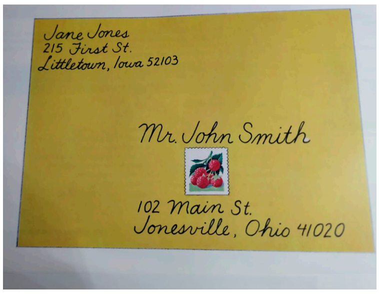

Change to
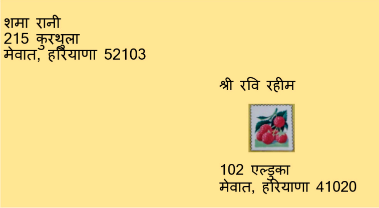
</td></tr>
</table>

Stanford Binet Item Book 2

- **Section: Verbal Level 3: Quantitative Reasoning**

Item No.	Item and Item Description	Change required/not required reason
Items 1 to 6	**Item 5: Janice had 5 books. She gave one to her friend, Linda. How many books did Janice have left?**	**Item 5: ~~Janice~~ Jamila had 5 books. She gave one to her friend, Linda. How many books did ~~Janice~~ Jamila have left?**

Stanford Binet Item Book 2

- **Section: Verbal Level 4: Fluid Reasoning**

Item No.	Item and Item Description	Change required/not required reason
Items 1 to 3	**Activity: Verbal Absurdities**<br><br>**The child has to point out silly or impossible aspects of each statement**<br><br>**Item 1: When Lewis gets dressed in the morning, he puts his boots on first, then his pants, shirt, and socks.**<br><br>**Item 2: Mary broke her leg yesterday, but today she is feeling better, so she skipped all the way home.**<br><br>**Item 3: One day we say several icebergs that had been entirely melted by the warmth of the Caribbean Sea**	**Suggested Changes**<br><br>**Item 1: When ~~Lewis~~ Latif gets dressed in the morning, he puts his boots on first, then his pants, shirt, and socks.**<br><br>**Item 2: ~~Mary~~ Mahira broke her leg yesterday, but today she is feeling better, so she skipped all the way home.**<br><br>**Item 3: One day we say several icebergs that had been entirely melted by the warmth of the ~~Caribbean Sea~~ Indian Ocean.**

Stanford Binet Item Book 2

- **Section: Verbal Level 4: Quantitative Reasoning**

**Item No.**	**Item and Item Description**	**Change required/not required reason**
Items<br>**1 to 6**	**Activity: Quantitative Reasoning**<br><br>**Item 4: If the sand in the small box weighs 10 pounds, how much des the sand in the large box weighs?**<br>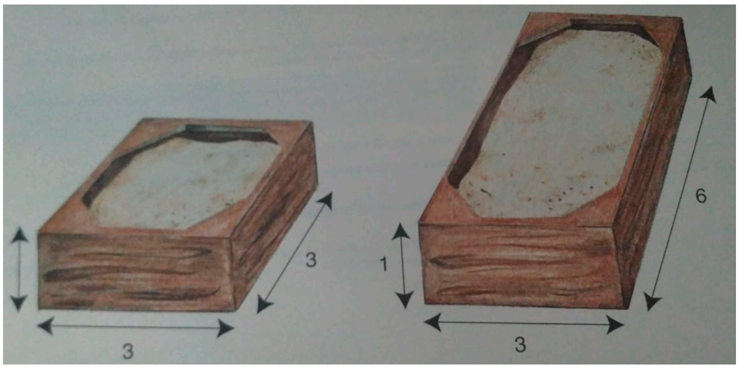<br>**Item 5: A man who weighs 150 pounds wants to reduce his weight to 130 pounds. If he lost 2 pounds a week, how many weeks would it take him to reach his goal?**<br><br>A man who weighs 150 pounds wants to reduce his weight to 130 pounds. If he lost 2 pounds a week, how many weeks would it take him to reach his goal?	Suggested Changes<br><br>**Item 4: If the sand in the small box weighs 10 ~~pounds~~ kilograms (kgs), how much des the sand in the large box weighs?**<br><br>**Item 5: A man who weighs 150 ~~pounds~~ kilograms (kgs) wants to reduce his weight to 130 ~~pounds~~ kilograms (kgs). If he lost 2 ~~pounds~~ kilograms (kgs)a week, how many**

		**weeks would it take him to reach his goal?**

Wechsler Intelligence Scale for Children IV (WISC IV) India adaptation version

	**Item and Item Description**	**Change required/not required reason**
**23**	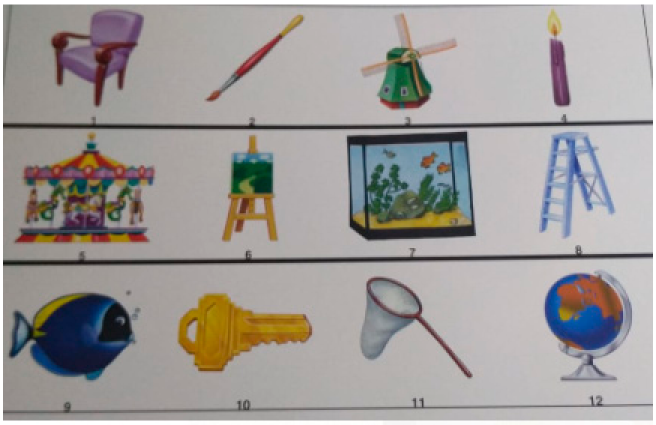	The child is required to figure out pictures (3,5,12) indicate objects that rotate/spin 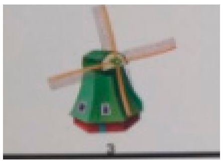 Change to 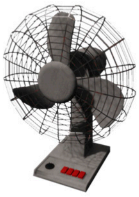 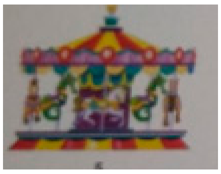 Change to 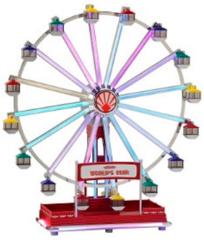

**25**	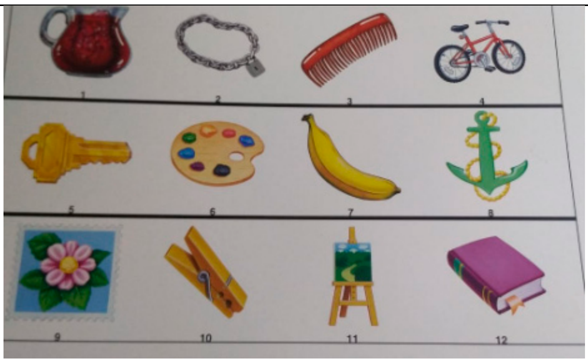	The child is required to figure out pictures (2,8,10) indicate objects that keep things in place<br>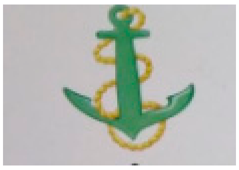<br>Change to<br>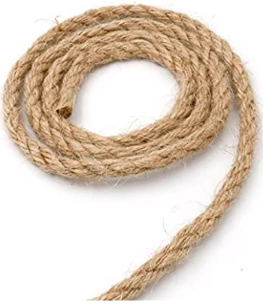
**27**	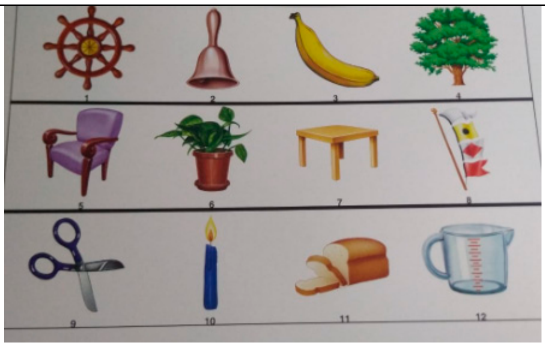	The child is required to figure out pictures (2,8,10) indicate objects that move<br>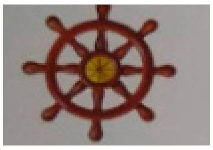<br>Change to<br>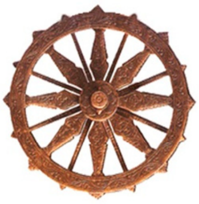

1. Subtest Letter-Number Sequencing

**Item No.**	**Item and Item Description**	**Change required/not required reason**
Items 1 to 10	**The child is read a sequence of numbers and letters and recalls the numbers in ascending order and the letters in alphabetical order.**<br><br>**Item 1**<br>• **Trail 1: A - 3**<br>• **Trail 2: B - 1**<br>• **Trial 3: 2 – C**<br><br>**Item 10**<br>• **Trail 1: 4 – B – 8 – R- 1 – M – 7 – H**<br>• **Trail 2: J – 2 – U – 8 – A – 5 – C – 4**<br>• **Trial 3: 6 – L – 1 – Z – 5 – H – 2 – W**	We will ask the child to recite the English alphabets A to Z. In the event that the child is not confident with the English letters the first 26 letters of Hindi/Urdu will be used in place of the English alphabets.

2. Subtest Comprehension

**Item No.**	**Item and Item Description**	**Change required/not required reason**
**Items 1 to 21**	**The child answers questions based on his or her understanding of general principles and social situations.**<br><br>**Items:**<br>**1. Why do people brush their teeth?**<br>**2. Why should people eat green vegetables?**<br>**3. What are you supposed to do if you find someone's wallet or purse in a shop?**<br>**4. Why do cars have seatbelts?**<br>**5. Why is it important for the police to wear uniforms?**<br>**6. Tell me some reasons that you should turn off lights when no one is using them.**<br>**7. What should you do if you see thick smoke coming from the window of your neighbour's house?**<br>**8. What is the thing to do if a boy or girl much smaller than yourself starts a fight with you?**<br>**9. What are the advantages of exercising and being active?**<br>**10. Why is it important to apologize when you know you've hurt someone?**	The participant will be asked these questions in Hindi.<br><br>Item 4: ~~**Why do cars have seatbelts?**~~<br>**Change to:**<br>**Why should we wear helmets when on a motorcycle/scooter?**

	11. Why is it important for the government to inspect mid-day meals before it is served in schools?<br>12. What are the advantages of having public libraries?<br>13. Why do doctors take up higher studies after treating patients for several years?<br>14. Tell me some advantages of getting the news from a newspaper rather than from a television news program?<br>15. Why should a promise be kept?<br>16. Why is freedom of speech important in democracy?<br>17. Why is it important to grant authors copyrights on books and inventors patents on inventions?<br>18. Why do we put stamps on letters?<br>19. Why is it important to keep one company from owning all of the newspapers and radio and TV stations in a single town or city?<br>20. What are some problems with rapid changes in science and technology?<br>21. How do communication tools (such as TV, radio and internet) threaten dictatorships?	Item 12: ~~**What are the advantages of having public libraries?**~~<br>**Change to:**<br>**What rea the advantages of a library?**

3. Subtest Arithmetic

Item No.	Item and Item Description	Change required/not required reason
Item 5	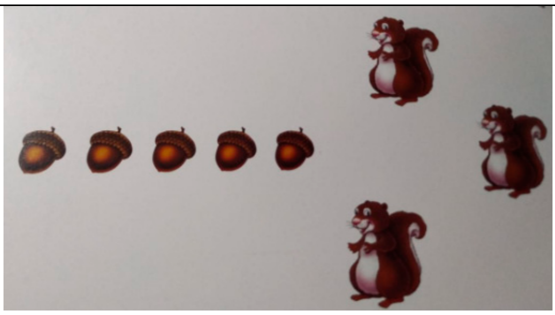	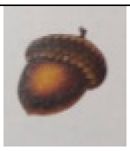<br>Change to<br>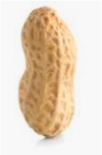

Supplementary Part S2

Cognitive

Subtest Item No	Cognitive English version	Translation
1	if [name] is crying and you pick him/her up does he/she calm down and stya calm almost every time he/she is picked up, some of the time or none of the time?	यदि [नाम] रो रहा है और आप उसे उठाते है तो क्या शांत हो जाता है? ;लगभग हमेशा जब उसको उठाते हो, कभी-कभी या कभी नहीं ?
6	When you are about to pick up (name) does [he/she] clearly show that [he/she] is looking forward to being picked up almost every time, some of the time or none of the time?	जैसे ही आप [नाम] को उठाने जाते हो, तो क्या वह स्पष्ट रूप से जतात है की वह उठाया जाना चाहते है ? लगभग हमेशा, कभी-कभी या कभी नहीं
15	When [insert child's name] is by [himself/herself], does [he/she] clearly look at [his/her] hands often, not often, or not at all?	जब [नाम] अपने आप रहता है, तो क्या वह स्पष्ट रूप से अपने हाथों को देखते है? अक्सर, अक्सर नहीं या बिलकुल भी नहीं
19	When [name] has something in his/her hand, does he/she purposely bang it on the furniture often, not often or not at all?	जब [नाम] के हाँथ में कुछ होता है, तो क्या वह जानबूझकर फर्नीचर पर उसको मरता /धमकाता है? अक्सर, अक्सर नहीं या बिलकल भी नहीं
26	When you show [name] a boook, does he/she look interested in the pictures almost every time, some of the time, or none of the time?	जब आप [नाम] को कोई किताब देखते हो, तो क्या वह चित्रों में रुचि /दिलचस्पी देखता/देखती है? लगभग हमेशा, कभी-कभी या कभी नहीं?
39	When playing, does [name] pretend to do things like drink from an empty cup?	जब [नाम] खेलता/खेलती है, तो क्या वह खली कप से कुछ पीने का नाटक करता/करती है?
41	When you read to [name], does he/she pay attention to the entire story almost every time, some of the time, or none of the time?	जब आप [नाम] को कोई कहानी सुनते/पढ़ते हो, तो क्या उसका ध्यान पूरी कहानी पर हमेशा होता है, कभी-कभी होता है या कभी नहीं होता
50	When playing, does [name] make-believe an object is something else, like pretending a ball is a piece of fruit? Can you give me some examples of other ways that he/she make-believes an object is something else?	खेलते समय, क्या [नाम] नाटक करता है की एक वस्तु कोई और चीज़ है, जैसे नाटक करना कि बॉल /गेंद एक फल का टुकड़ा है? क्या आप मुझे और कुछ उदाहरण दे सकते है जिसमे वह एक वास्तु को कुछ और मानता/मानती है
59	When playing, does [name] use imaginary object? Can you give me examples of him/her using imaginary objects?	खेलते समय, क्या [नाम] काल्पनिक वस्तुओं का उपयोग करता/करती है? क्या आप मुझे कुछ उदहारण दे सकती है

Receptive Communication

Subtest	Receptive Communication	
Item No.	English Version	Translation
5	Does [name] respond to your voice by doing things like turning his/her head or changing the look on his/her face almost every time, some of the time or none of the time?	क्या [नाम] आपकी आवाज़ को सुनकर अपना सर मोड़ता/मोड़ती है या उसके चेहरे पर कोई बदलाव आता है, लगभग हर बार, कभी-कभी या कभी नहीं?
6	When you are about to pick up [name], does he/she clearly show that he/she is looking forward to being picked up almost every time, some of the time or none of the time?	जब आप [नाम] को गोद में लेने वाले होते हैं, तो क्या वह स्पष्ट रूप से दिखता/दिखती है की वह आपकी गोद में आना चाहता/चाहती है, लगभग हर बार, कभी-कभी या कभी नहीं?
11	When [name] has something in his/her hand, does he/she purposely bang it on the furniture often, not often or not at all?	जब [नाम] के हाथ में कुछ होता है, तो क्या वह फ़र्नीचर पर जानबूझकर उस चीज़ को धमकाता/ धमकाती है अधिकतर, कभी-कभी या बिल्कुल नहीं?
12	Does [name] respond to you when you call his/her name by turning his/her without you touching him/her almost every time, some of the time or none of the time?	जब आप [नाम] का नाम पुकारते हो, उसे छुए बिना, तो क्या वह आपकी ओर मुड़ कर देखता/देखती है, लगभग हर बार, कभी-कभी या कभी नहीं?
13	When you say the name of a familiar object, does [name] look at the object you named often, not often or not at all?	जब आप किसी परिचित वस्तु का नाम लेते हैं, तो क्या [नाम] उस वस्तु को देखता/देखती है अधिकतर, कभी-कभी या बिल्कुल नहीं?
14	When you ask [name] to do things, like give a high five or blow a kiss, does he/she do what you ask without you showing him/her to do it almost every time, some of the time or none of the time	जब आप (नाम) को कुछ करने को कहते हैं जैसे हाथ से हाथ थपथपाना, या होठों को चुम्मा देने के लिए मोड़ना तो क्या वह कर पाता/पाती है, बिना दिखाए कि कैसे करना है, लगभग हर बार, कभी-कभी या कभी नहीं?
21	feed, comb and wipe	खाना खाना, कंघी करना और पोंछना
29	her, me, you, my, your (pronouns need translating)	उसकी, मैं, तुम/आप, मेरी, तुम्हारी/ आपकी

Expressive Communication

Subtest	Expressive Communication	
Item No.	English Version	Translation
1	When you play and talk with [name], does he/she make sounds, like gurgling, grunting and squealing often, not often or not at all?	जब आप खेलते या बात करते हैं (नाम) के साथ, क्या वह ख़ुशी की आवाजें निकालता/ निकालती है अधिकतर, कभी-कभी या बिल्कुल नहीं?
2	Does [name] smile or laugh when you or other adults look at or snuggle with him/her often, not often or not at all?	क्या (नाम) मुस्कुराता/मुस्कुराती या हंसता/हंसती है जब आप या अन्य व्यस्क लोग उसकी तरफ़ देखते हैं या उसके साथ आराम से बैठते हैं, अधिकतर, कभी-कभी या बिल्कुल नहीं?
3	Does [name] make noises other than crying to show you how he/she feels when he/she is happy or upset, like laughing or whining often, not often or not at all?	क्या (नाम) रोने की आवाज़ के इलावा और आवाज़ें निकालता/निकालती है दिखाने के लिए वह कैसा महसूस करता/करती है, जब ख़ुश होता/होती है या दुःखी होता/होती है जैसे हंसना या कराहना, अधिकतर, कभी-कभी या बिल्कुल नहीं?
4	Does [name] make noises or laugh when you speak to or snuggle with him/her often, not often or not at all?	क्या (नाम) आवाज़ें निकालता/ निकालती है या हंसता/हंसती है जब आप उससे बात करते हैं या उसके साथ आराम से बैठते हैं, अधिकतर, कभी-कभी या बिल्कुल नहीं?
5	When [name] makes sounds, do you hear him/her make sounds like ah, uh, ooh or eh often, not often or not at all?	जब (नाम) आवाजें निकालता /निकालती है तो क्या आपको आह, उह, ऊह, एह जैसे स्वर सुनाई देते हैं अधिकतर, कभी-कभी या बिल्कुल नहीं?
6	When you are not paying attention to [name], does he/she try to get your attention often, not often or not at all?	जब आप नाम की ओर ध्यान ना दे रहे हो तो क्या वह आपका ध्यान अपनी ओर खींचने की कोशिश करता करती है, अधिकतर, कभी-कभी या बिल्कुल नहीं?
7	When [name] makes sounds, do you hear him/her make sounds like da, ma, or bah often, not often or not at all?	जब (नाम) आवाजें निकालता /निकालती है तो क्या आपको दा, मा, बाह, जैसे स्वर सुनाई देते हैं अधिकतर, कभी-कभी या बिल्कुल नहीं?
8	When [name] makes sounds, do you hear him/her make sounds, like gaga, bababa, dada or mamama often, not often or not at all?	जब (नाम) आवाज़ें निकालता/निकलती है, तो क्या आप गा गा, बा बा बा, दादा या मा मा मा जैसी आवाज़ें सुनते हैं, अधिकतर, कभी-कभी या बिल्कुल नहीं?
9	When you play and talk with [name], do you see him/her use gestures, like shaking his/her head no or waving bye-bye often, not often or not at all?	जब आप (नाम) के साथ खेलते हैं या बात करते हैं, क्या आप उसे इशारे करते देखते हैं जैसे ना करने के लिए सिर हिलाना या बाई-बाई हाथ से करना, अधिकतर, कभी-कभी या बिल्कुल नहीं?
10	When you talk to [name], does he/she jabber back at you often, not often or not at all?	जब आप (नाम) से बात करते हैं तो क्या वह आपसे गपशप करता/करती है अधिकतर, कभी-कभी या बिल्कुल नहीं?

11	When [name] sees people or he/she plays with toys, does he/she name or label them often, not often or not at all?	जब (नाम) लोगों को देखता/देखती है या खिलौनों से खेलता/खेलती है, तो क्या उन्हें वह नाम या पहचान देता/देती है, अधिकतर, कभी-कभी या बिल्कुल नहीं?
12	When [name] wants you to look at something, does he/she point to the object or hand it to you without you asking him/her about the object often, not often or not at all?	जब (नाम) आपको कुछ चीज़ दिखाना चाहता/चाहती है, तो क्या वह उस ओर इशारा करता/ करती है या आपको पकड़ा देता/देती है बिना आपके उस चीज़ के बारे में पूछे अधिकतर, कभी-कभी या बिल्कुल नहीं?
13	Does [name] repeat the words you say to him/her back to you often, not often or not at all?	क्या (नाम) वो शब्द दौहराता/दौहराती है जो आप उसे कहते हैं, अधिकतर, कभी-कभी या बिल्कुल नहीं?
14	When [name] plays, does he/she try to start a game with you, like peek-a-boo often, not often or not at all?	जब (नाम) खेलता/खेलती है तो क्या वह किसी चीज़ के पीछे छुपने और ढूंढने का खेल खेलता/खेलती है, अधिकतर, कभी-कभी या बिल्कुल नहीं?
15	When [name] talks, does he/she use the right words to talk about objects he/she is playing with or to describe what is going on often, not often or not at all?	जब (नाम) बात करता/करती है तो क्या वह सही शब्द इस्तेमाल करता/करती है, उन चीज़ों के लिए जिनके साथ वो खेलता/खेलती है, या क्या हो रहा है, अधिकतर, कभी-कभी या बिल्कुल नहीं?
17	When new people talk with [name], do they understand the words he/she says at least half/50% of the time, less than half/50% of the time, or none of the time, often, not often or not at all?	जब नए लोग (नाम) से बात करते हैं, क्या वह 50% समय, 50% समय से कम या बिल्कुल नहीं उसके शब्दों को समझ पाते हैं, अधिकतर, कभी-कभी या बिल्कुल नहीं?
19	When you play and talk with [name], does he/she gesture and speak at the same time to express himself/herself often, not often or not at all?	जब आप (नाम) के साथ खेलते हैं या बात करते हैं, तो क्या वह बोलने के साथ-साथ इशारे करता/करती है अपनी बात को समझाने के लिए, अधिकतर, कभी-कभी या बिल्कुल नहीं?
21	When you ask [name] a yes/no question, does he/she answer you by saying the words yes or no almost all the time, some of the time or none of the time?	जब आप (नाम) से हां/ना वाला प्रश्न पूछते हैं,तो क्या वह आपको हां या ना में जवाब देता/देती है लगभग हर बार, कभी-कभी या कभी नहीं?
22	Does [name] repeat two-repeat two-word sentences back to you, like Mama go often, not often or not at all?	क्या (नाम) दौहराता/दौहराती है दो शब्दों वाले वाक्य, जैसे, मां जाओ, अधिकतर, कभी-कभी या बिल्कुल नहीं?
23	When you play and talk with [name], does he/she say sentences with two or more words often, not often or not at all?	जब आप (नाम) के साथ खेलते हैं या बात करते हैं क्या वह दो शब्दों या अधिक शब्दों वाले वाक्य बोलता/बोलती है, अधिकतर, कभी-कभी या बिल्कुल नहीं?
24	When new people talk with [name], do they understand the sentences he/she says 75% of the time, only half/50% of the time, or less than half/50% of the time?	जब नए लोग (नाम) से बात करते हैं क्या वह 75% समय या 50% समय या 50% समय से कम उसके वाक्य समझ पाते हैं?
25	When you play and talk with [name], does he/she say sentences with three or more words often, not often or not at all?	जब आप (नाम) के साथ खेलते हैं या बात करते हैं, तो क्या वह तीन शब्दों या उससे अधिक शब्दों वाला वाक्य बोलता/बोलती है, अधिकतर, कभी-कभी या बिल्कुल नहीं?

26	When you play and talk with [name], does he/she say pronouns, like me, **I**, he, she, her or him often, not often or not at all?	जब आप (नाम के साथ खेलते हैं या बात करते हैं तो क्या वह मुझसे, मैं. वह, उसकी या उसे ऐसे सर्वनामों का उपयोग करता/ करती है, अधिकतर, कभी-कभी या बिल्कुल नहीं?
37	When you play and talk with [name], does he/she say sentences with four or five words often, not often or not at all?	जब आप (नाम) के साथ खेलते हैं या बात करते हैं तो क्या वह चार या पाँच शब्दों वाले वाक्य बोलता/बोलती है, अधिकतर, कभी-कभी या बिल्कुल नहीं?

Fine Motor

Subtest	Fine Motor	
Item No.	English Version	Translation
4	Can [name] successfully hand(s) in his/her mouth almost..	क्या नाम पूरी तरह अपना अपने हाथ मुंह में डाल लेता लेती है या कुछ हद तक
5	When [name] has something in his/her hand, is he/she able to hold onto it for at least 5 seconds..	नाम के हाथ में कोई चीज होती है तो क्या वह उसे कम से कम 5 सेकंड तक पकड़े रख सकता सकती है
8	When [name] is not holding something, are his/her hands open..	जब नाम हाथ में कुछ नहीं पकड़े होता होती तो क्या उसके हाथ खुले होते हैं
11	Is [name] able to successfully grab things that hand, like this ring (point to ring)..	क्या नाम पूरी तरह अपने हाथ से चीजें झपट सट्टा सकती है जैसे की अंगूठी अंगूठी की ओर इशारा कीजिए
12	When things like this block (point to block) are placed in front of [name], is he/she able to reach for and touch them..	जब चीज है जैसे कि ब्लॉक ब्लॉक की ओर इशारा करें नाम के सामने रखे जाते हैं क्या वह उस तक पहुंच सकता सकती है और उसे छू सकता सकती है
17	When you give [name] small foods, like *peas, puffs* or *raisins*, is he/she able to successfully pick them up with his/her thumb and finger and put them in his/her mouth..	नाम को छोटी-छोटी खाने की चीजें देते हैं जैसे मटर या किशमिश क्या वह अच्छी तरह अपनी उंगली और अंगूठे से उन्हें उठा पाता पाती है और मुंह में डाल पाता पाती है
20	Is [name] able to successfully turn the pages of a book one at a time..	नाम अच्छी तरह किताब का 11 पृष्ठ पलट पाता पाती है

Gross Motor

Subtest	Gross Motor	
Item No.	English Version	Translation
10	Can [name] successfully roll from his/her stomach to his/her back almost every time he/she tries, some of the time or none of the time?	क्या (नाम) सफ़लतापूर्वक अपने पेट से अपनी पीठ पर मुड़ जाता/जाती है हर बार जब कोशिश करता/करती है, लगभग हर बार, कभी- कभी या कभी नहीं?
22	Does[name] grab his/her feet or put them in his/her mouth almost every time he/she tries, some of the time or none of the time?	क्या (नाम) अपने पांव को झपटता/झपटती है और अपने मुंह में डाल सकता/सकती है, लगभग हर बार, कभी- कभी या कभी नहीं?
23	Can [name] successfully roll from his/her back to his/her stomach almost every time he/she tries, some of the time or none of the time?	क्या (नाम) अपनी पीठ से अपने पेट पर सफ़लतापूर्वक मुड़ जाता/जाती है, हर बार जब वह कोशिश करता/ करती है, लगभग हर बार, कभी- कभी या कभी नहीं?
26	Does [name] crawl on his/her hands and knees? / When [name] wants to move from one place to another, can he/she do the army or commando crawl or crawl on his/her stomach for at least 3 feet, almost every time he/she tries, some of the time or none of the time?	क्या (नाम) अपने हाथों और घुटनों पर क्रॉल करता/करती है? जब (नाम) एक जगह से दूसरी जगह जाना चाहता/चाहती है, तो क्या वह आर्मी क्रॉल या कमांडो क्रॉल 3 फ़ीट तक कर सकता/सकती है, लगभग हर बार, कभी- कभी या कभी नहीं?
27	When [name] wants to get up and start crawling, can he/she go from a sitting to a crawling position without tipping over or falling down almost every time he/she tries, some of the time or none of the time?	जब (नाम) बैठे हुई मुद्रा से उठकर क्रॉल करना चाहता है तो क्या वह बिना संतुलन खोए या गिरे ऐसा कर पाता/पाती है, लगभग हर बार, कभी- कभी या कभी नहीं?
29	When [name] wants to move from one place to another, can he/she crawl on his/her hands and knees for at least 5 feet almost every time he/she tries, some of the time or none of the time?	जब (नाम) एक जगह से दूसरी जगह जाना चाहता/चाहती है, तो क्या वह 5 फ़ीट तक अपने हाथों और घुटनों के सहारे क्रॉल कर पाता/पाती है, लगभग हर बार, कभी- कभी या कभी नहीं?
30	Can [name] successfully pull up on furniture to a standing position almost every time he/she tries, some of the time or none of the time?	क्या (नाम) सफ़लतापूर्वक ख़ुद को ठीक तरह किसी कुर्सी, टेबल, आदि को पकड़कर खड़ा/खड़ी कर सकता/सकती है, लगभग हर बार, कभी- कभी या कभी नहीं??
32	When [name] wants to move from one place to another, can he/she walk sideways while holding onto the furniture almost every time he/she tries, some of the time or none of the time?	जब (नाम) एक जगह से दूसरी जगह जाना चाहता/चाहती है, क्या वह कुर्सी, टेबल, आदि को पकड़कर तिरछा चल पाता/पाती है, लगभग हर बार, कभी- कभी या कभी नहीं?
37	Is [name] able to successfully throw a ball forward almost every time he/she tries, some of the time, or none of the time?	क्या (नाम) हर बार, कभी-कभी या कभी नहीं गेंद को सफ़लतापूर्वक सामने फेंक सकता/सकती है जब भी वह कोशिश करता/करती है, लगभग हर बार, कभी- कभी या कभी नहीं?
44	Does [name] jump from the bottom step and land without stumbling or falling down almost every time he/she tries, some of the time, or none of the time?	क्या (नाम) सबसे निचली सीढ़ी से कूदकर ज़मीन पर पहुंचता/पहुंचती है बिना संतुलन खोए या गिरे; लगभग हर बार, कभी-कभी या कभी नहीं, जब भी वह कोशिश करता/ करती है?

48	Is [name] able to jump off the floor with both feet in the air at the same time almost every time he/she tries, some of the time, or none of the time?	क्या (नाम) ज़मीन से छलांग लगाते समय दोनों पैर इकट्ठे उठाता/उठाती है, लगभग हर बार, कभी-कभी या कभी नहीं?

Social Emotional

Subtest Item No	Social Emotional Scale English version	Translation
1	Takes a calm an enjoyable interest in most sounds	शांत और मज़ेदार दिलचस्पी लेता/लेती है अधिकतर आवाज़ों /स्वरों में।
2	You can easily get your child's attention without having to be very dramatic	आप अपने बच्चे का ध्यान आसानी से अपनी और खींच सकते हैं बिना अधिक नाटकीय हुए ।
3	Takes a calm and enjoyable interest in most sights, including colorful or bright things	शांत और मज़ेदार दिलचस्पी लेता/लेती है जो दिखाई देता है, रंगीन और चमकदार चीजें इसमें शामिल हैं।
4	You can easily get your child to look at things without them being very bright or colorful	आप अपने बच्चे को आसानी से उन चीज़ों की तरफ़ देखने को प्रभावित कर सकते हैं,जो ज़्यादा चमकदार और रंगीन ना हों ।
5	Calmly enjoys touching or being touch by different things	शांतिपूर्वक मज़ा लेता/लेती है चीज़ों को छूने में या चीज़ें जब उसे छूती हैं ।
6	You can easily get your child to respond to your touch without having to touch your child firmly to get his/her attention.	आप अपने बच्चे की प्रतिक्रिया आसानी से अनुभव कर सकते हैं जब आप उसे छूते हैं । आपको बहुत ज़ोर से नहीं छूना पड़ता, उसका ध्यान अपनी ओर खींचने के लिए ।
7	Likes to be swung around, danced with while in your arms, or quickly lifted up in the air.	बच्चा पसंद करता है आपकी बाहों में झूलाना, बाहों में नाचना या जल्दी से हवा में ऊपर उठाना ।
8	You can easily get your child's attention by approaching him/her or moving him/her slowly	आप अपने बच्चे का ध्यान अपनी और आसानी से खींच सकते हैं, उसकी ओर आते हुए या उससे धीरे से हिलाते हुए ।
9	You can help your child calm down	आप अपने बच्चे की मदद कर सकते हैं शांत होने में ।
10	Looks at interesting sights, such as your face or a toy.	दिलचस्प चीजों को बच्चा देखता है, जैसे कि आपका चेहरा या कोई खिलौना ।
11	Looks at or turns towards interesting sounds	देखता/देखती है या मुड़ता/मुड़ती है दिलचस्प आवाज़ों की ओर ।
	Stop here if your child is 0-3 months old	
12	Seems happy or pleased when he/she sees a favorite person (e.g. looks or smiles, makes sounds, or moves arms in a way that expresses joy or delight)	ख़ुश नजर आता/आती है जब अपने सबसे मनपसंद जन को देखता/देखती है, उदाहरण: देखता/ देखती है, मुस्कुराता/मुस्कुराती है,आवाज़ें निकालता/ निकालती है या अपनी बाहें इस प्रकार हिलाता/हिलाती है कि ख़ुशी प्रकट हो।

13	Responds to people talking or playing with him/her by making sounds or faces (eg happy sounds or a curious or annoyed look)	प्रतिक्रिया ज़ाहिर करता/करती है जब उससे लोग बात करते हैं या खेलते हैं, आवाज़ें निकालकर या मुंह से अलग-अलग शक्लें बनाकर। उदाहरण: ख़ुशी की आवाज़ें, कुछ उत्सुकता वाली शक्ल या गुस्से वाली शक्ल।
	Stop here if your child is 4-5 months old	
14	reaches for or points at things, or makes distinct sounds to show you what he/she wants for example reaches out to be picked up or points at a toy	चीज़ों की ओर जाता/जाती है या उंगली से चीज़ों की ओर इशारा करता/करती है या स्पष्ट आवाज़ निकालता/निकालती है, आपको बताने के लिए कि उसे क्या चाहिए। उदाहरण: हाथों को ऊपर करता/ करती है ताकि आप उसे उठा लें या किसी खिलौने की तरफ़ इशारा करता/करती है।
15	exchanges, two or more smiles, other looks, sounds or actions, for example, reaching, giving or talking with a favorite person	दो या दो से ज़्यादा बार मुस्कुराता/मुस्कुराती है आपके साथ, अन्य भाव, स्वर या क्रियाएं भी व्यक्त करता/करती है । उदाहरण: आगे बढ़ना, कुछ देना या अपने सर्वप्रिय जन से बात करना ।
	Stop here if your child is 6-9 months old	
16	shows you that he she understands your actions or gestures by making an appropriate gesture in return for example makes a funny face back at you, looks at something you point to stop doing something when you shake your head and use a form voice just say no or smiles and does more of something when you not with the big smile and say yes	दिखाता/दिखाती है कि वह आपकी क्रियाएं या इशारे समझता/समझती है, आपकी तरफ़ उचित इशारे करके । उदाहरण: आपकी और हंसाने वाली शक्ल बनाती/बनाता है, आपकी हंसाने वाली शक्ल देख कर किसी चीज़ की ओर देखता/देखती है, जब आप उसकी तरफ़ इशारा करते हैं किसी क्रिया काम को बंद करने का, जब आप ना में सिर हिलाते हैं, कड़क आवाज़ इस्तेमाल करते हैं या ना कहते हैं, क्या वह हंसता/हंसती है और वही क्रिया और अधिक करता/करती है जब एक बड़ी मुस्कुराहट के साथ हां में सिर हिलाते हैं और हां कहते हैं ।
17	. uses many consecutive actions in a back and forth way to show you what he or she wants, or to have fun with you, for example, smiles, reaches out for a hug. And when you hug, takes your hat pulls it on his/her forehead and smiles proudly or takes your hand leads you to the refrigerator, tugs on the handle and after you open it points to something he likes such as food, a bottle of juice or milk.	एक साथ कई क्रियाएं आगे पीछे करता/करती है आपको दिखाने के लिए कि उसे क्या चाहिए या आपके साथ मज़ाक करने के लिए। उदाहरण: मुस्कुराकर आपकी ओर, जप्फी के लिए बाहें बढ़ाता/बढ़ाती है। जब आप जप्फी देते हैं तो आप की टोपी अपने सर पर खींच लेता/लेती है और गर्व से मुस्कुराता/मुस्कुराती है या आपका हाथ पकड़कर फ्रिज की ओर ले जाता/जाती है, हैंडल को छूता/छूती है और जब आप फ्रिज खोलते हैं तो अपनी मनपसंद चीज़ की ओर इशारा करता/करती है जैसे खाना, जूस की बोतल या दूध।

Stop here if your child is 10-14 months old		
18	copies or imitates many of your sounds, words or actions while playing with you. For example, if you make funny faces and sounds he she copies them	आपकी अधिकतर आवाज़ों, शब्दों या क्रियाओं को दोहराता/दोहराती है या आपकी नकल उतारता/उतारती है, आपके साथ खेलते समय। उदाहरण: यदि आप मज़ाकिया शक्लें बनाते हैं या आवाज़ें निकालते हैं तो वह आपकी नकल उतारता/उतारती है।
19	searches for something he she wants by looking or getting you to look for it	कुछ ढूंढता/ढूंढती है जो उसे चाहिए आपकी ओर देखते हुए या आपसे ढुंढवाते हुए।
20	shows you what he or she wants or needs we're using a few actions in a row, for example, leads you by the hand to open a door and then touches, or bangs on the door	दिखाता/दिखाती है उसे क्या चाहिए या ज़रूरत है। हम कुछ क्रियाएं लगातार उपयोग कर रहे हैं। उदाहरण: आपको हाथ पकड़ कर दरवाज़ा खोलने के लिए दरवाज़े की ओर ले जाता/ जाती है और पहुंचते ही दरवाज़े को छूता/छूती है ज़ोर से ठकठकाता/ठकठकाती है।
21	uses words or tries to use words when people talk with or play with him/her	शब्दों का प्रयोग करता/करती या कोशिश करता/करती है जब लोग उससे बात करते हैं या उसके साथ खेलते हैं।
Stop here if your child is 15-18 months old		
22	copies or imitates familiar make believe play for example feeds, or hugs, a doll	नकल करता/करती है जान पहचान के काल्पनिक खेल जैसे गुड़िया को खिलाना या जप्फी मारना।
23	tells you what he or she wants with one or a few words like: juice, open, kiss	आपको बताता/बताती है एक शब्द या कुछ शब्दों में कि उसे क्या चाहिए, जैसे जूस. खोलो, चुम्मा।
24	shows you he/she understands you. Your simple verbal wish for example, please show me your toy.	दिखाता/दिखाती है कि वह आपको समझता/समझती है, जैसे आपकी साधारण मौखिक कामना, आप कृपया मुझे अपना खिलौना दिखाएं।
Stop here if your child is 19-24 months old		
25	plays make believe, for example feeds doll, plays house or pretends to be TV or movie character with you or others	काल्पनिक खेल खेलता/खेलती है जैसे गुड़िया को खिलाना, घर-घर खेलना या कोई टीवी का या फिल्म का पात्र बनकर आप से या अन्य जन से खेलना।
26	uses words or pictures to tell you what he/she is interested in, for example, see truck	शब्दों का या तस्वीरों का उपयोग करके बताना/बताती है कि उसकी रुचि किसमें है जैसे, देखो ट्रक।
27	uses words with one or more peers	शब्दों का उपयोग करता/करती है हमउम्र बच्चों के साथ।
28	uses words or pictures to show what he/she likes or dislikes, for example, want that, no want	शब्दों का या तस्वीरों का उपयोग करके बताता/बताती है कि उसे क्या पसंद है, जैसे चाहिए वह, नहीं चाहिए।

Stop here if your child is 25-30 months old

29	Plays make believe with one or more peers	अपने हमउम्र बच्चों के साथ काल्पनिक खेल खेलता/खेलती है।
30	Plays make believe with you or others, where the story makes sense for example has the best-go visit grandmother and then have a big lunch	आपके साथ या अन्य जन के साथ काल्पनिक खेल खेलता/खेलती है, जहां कहानी सार्थक होती है । उदाहरण: सबसे अच्छा खेल; दादी मां/नानी मां को मिलने जाना, और फिर बहुत बड़ा भोजन करना ।
31	Uses phrases or sentences with you to ask a question about something he or she wants to do, for example, Mommy go out? What you doing outside? Play?	शब्दों और वाक्यों के माध्यम से वह आपसे प्रश्न पूछता/पूछती है जो वह करना चाहता/ चाहती है उस विषय में, जैसे, मां बाहर जा? आप क्या बाहर कर रहे? खेलना?
32	can explain why he/she wants something or wants to do something eg. Why do you want the juice? Because I'm thirsty	समझा सकता/सकती है क्यों उसे वह चीज़ क्यों चाहिए और क्यों वह, वह क्रिया करना चाहता/ चाहती है। जैसे, तुम्हें जूस क्यों चाहिए, क्योंकि मैं प्यासा/प्यासी हूं।
33	Describes his/her feelings to explain why he or she is doing something, or wants something. For example, because I'm happy/sad/ excited	अपनी भावनाओं को व्यक्त करता/करती है यह समझाने के लिए कि वह, वह क्रिया क्यों कर रहा/रही है या उसे वह चीज़ क्यों चाहिए उदाहरण: क्योंकि मैं ख़ुश/ दुःखी/ उत्सुक हूं।
34	Plays make believe with peers, as well as adults with the story makes sense and he has many parts to it. For example, the children go to school, do work, have lunch, and meet an elephant on the way home.	काल्पनिक खेल खेलता/खेलती है हमउम्र बच्चों के साथ और बड़ों के साथ, जिसमें कहानी में अर्थ निकलता है और उसके कई हिस्से होते हैं। उदाहरण: बच्चे स्कूल जाते हैं, काम करते हैं, खाना खाते हैं और घर आते समय एक हाथी से मिलते हैं।
35	has conversations with adults and peers, that makes sense with four or more, back and forth, exchanges, about a variety of topics for example, feelings, foods, bedtimes, friends, or school	बड़ों और हमउम्र बच्चों के साथ वार्तालाप करता/करती है जिसका अर्थ निकलता है और बातचीत एक दूसरे से चार या ज़्यादा बार होती है, अलग-अलग विषयों पर। उदाहरण: भावनाएँ, व्यंजन, सोने का समय, मित्र या स्कूल।

Stop here if your child is 31-42 months old
